# Non-Contact Heart Rate Variability Monitoring with FMCW Radar via a Novel Signal Processing Algorithm

**DOI:** 10.3390/s25175607

**Published:** 2025-09-08

**Authors:** Guangyu Cui, Yujie Wang, Xinyi Zhang, Jiale Li, Xinfeng Liu, Bijie Li, Jiayi Wang, Quan Zhang

**Affiliations:** School of Information and Communication Engineering, North University of China, Taiyuan 030051, China; 13848982614@163.com (G.C.); wangyujie2512@163.com (Y.W.); 15340650581@163.com (X.Z.); 15340682084@163.com (J.L.); lxf18735060428@163.com (X.L.); sz202405009@st.nuc.edu.cn (B.L.); 18295706140@163.com (J.W.)

**Keywords:** heart rate variability (HRV), millimeter-wave (mmWave) radio, non-contact monitoring, spectral sparse separation algorithm

## Abstract

Heart rate variability (HRV), which quantitatively characterizes fluctuations in beat-to-beat intervals, serves as a critical indicator of cardiovascular and autonomic nervous system health. The inherent ability of non-contact methods to eliminate the need for subject contact effectively mitigates user burden and facilitates scalable long-term monitoring, thus attracting considerable research interest in non-contact HRV sensing. In this study, we propose a novel algorithm for HRV extraction utilizing FMCW millimeter-wave radar. First, we developed a calibration-free 3D target positioning module that captures subjects’ micro-motion signals through the integration of digital beamforming, moving target indication filtering, and DBSCAN (Density-Based Spatial Clustering of Applications with Noise) clustering techniques. Second, we established separate phase-based mathematical models for respiratory and cardiac vibrations to enable systematic signal separation. Third, we implemented the Second Order Spectral Sparse Separation Algorithm Using Lagrangian Multipliers, thereby achieving robust heartbeat extraction in the presence of respiratory movements and noise. Heartbeat events are identified via peak detection on the recovered cardiac signal, from which inter-beat intervals and HRV metrics are subsequently derived. Compared to state-of-the-art algorithms and traditional filter bank approaches, the proposed method demonstrated an over 50% reduction in average IBI (Inter-Beat Interval) estimation error, while maintaining consistent accuracy across all test scenarios. However, it should be noted that the method is currently applicable only to scenarios with limited subject movement and has been validated in offline mode, but a discussion addressing these two issues is provided at the end.

## 1. Introduction

Real-time monitoring of human physiological states is crucial for modern health management and disease prevention. Heart rate variability (HRV), which reflects autonomic nervous system regulation and cardiac function, has been extensively applied in early diagnosis of cardiovascular diseases and long-term health assessment [1,2]. However, conventional HRV measurement methods rely on contact sensors such as an electrocardiogram (ECG) or photoplethysmography (PPG). Despite their accuracy in signal acquisition, these contact methods may cause discomfort, skin allergies, and are unsuitable for vulnerable populations (e.g., patients with skin infections or burns). Consequently, non-contact monitoring technologies have emerged as a significant research focus.

Among non-contact technologies, Frequency-Modulated Continuous Wave (FMCW) millimeter-wave radar offers significant advantages, including high resolution, real-time capability, and obstacle penetration. Compared to camera-based solutions, FMCW radar systems better mitigate privacy concerns [3]. Recent advancements in millimeter-wave radar have improved both signal processing and target detection methodologies. Early research utilized singular value decomposition (SVD), matched filtering, and related techniques to isolate heartbeat signals from confounding factors like respiration. However, these conventional methods often yield insufficient extraction accuracy. While deep learning and iterative optimization techniques have enhanced HRV parameter precision, their generalization capability remains limited by insufficient validated datasets.

To address these limitations, we propose a novel three-dimensional human positioning and HRV extraction iterative optimization method (Second-Order Spectral Sparse Separation Algorithm Using Lagrangian Multipliers) using FMCW millimeter-wave radar. Our method achieves precise 3D targeting through positional and signal enhancement techniques, combining beamforming with Moving Target Indication (MTI) for robust detection. Second, we establish mathematical models for respiratory and heartbeat signals, analyzing their characteristic differences to provide a theoretical foundation for iterative optimization algorithms. Finally, we introduce a Second-Order Spectral Sparse Separation Algorithm Using Lagrangian Multipliers, which reduces parameter extraction errors compared to conventional methods.

This investigation has yielded encouraging results in both theoretical modeling and experimental validation, providing a viable new approach for non-contact physiological state detection. The remainder of the manuscript is organized as follows: Section 2 reviews related work and evaluates the advantages and disadvantages of existing methods; Section 3 elucidates the principles of radar vital sign detection and the detailed implementation of each algorithm step; Section 4 presents the experimental setup and analysis of results; Section 5 discusses system limitations and potential avenues for future improvement.

## 2. Related Work

The evolution of non-contact heart rate variability (HRV) detection technology has progressed substantially from conventional signal processing methodologies to advanced intelligent data analysis techniques. The field has witnessed continuous refinement, whether employing classical filtering, matching algorithms, and two-dimensional positioning techniques characteristic of conventional methods, or intelligent signal processing solutions leveraging neural networks and deep learning architectures, with significant advancements in key areas such as signal separation, noise suppression, and real-time detection. These methodologies have exhibited considerable potential in diverse practical application scenarios, encompassing medical health monitoring, sleep analysis, smart home environments, and driving safety systems.

### 2.1. Traditional Methods

The development of traditional algorithms has been primarily reflected in the continuous iteration of hardware platforms and signal processing methods, with the core aim of effectively separating and accurately extracting heartbeat and respiratory signals through various techniques, including filtering, differential enhancement, spectral estimation, and two-dimensional positioning technologies. From the initial use of ultrasonic transducers to build non-contact HRV measurement systems for evaluating long-term monitoring uncertainty [4], to utilizing 24 GHz continuous-wave Doppler radar that combines frequency and time domain analysis to achieve high-precision heartbeat interval estimation [5], these pioneering studies laid the theoretical and practical foundation for non-contact HRV detection.

To further overcome the challenges of environmental interference and respiratory harmonic interference, the detectability of heartbeat signals was significantly improved by introducing differential enhancement technology [6]. Meanwhile, the development of FMCW radar technology has facilitated the realization of long-distance and non-intrusive monitoring, with applications encompassing remote detection of human vital signs, separation of cardiopulmonary activities, and multi-scenario monitoring during sleep [7].

In addressing multi-target detection and multi-path propagation problems in complex indoor environments, multiple-input multiple-output (MIMO) radar with digital beamforming (DBF) technology, singular value decomposition combined with matched filtering algorithms, and two-dimensional positioning with angle separation methods were successively proposed, enabling robust detection and precise positioning of multiple human targets’ vital signs [8,9,10]. Additionally, multi-user HRV systems based on millimeter-wave radio further improved detection accuracy through calibrated free target detection and signal decomposition [11], while spectral estimation methods and real-time evaluation algorithms facilitated high-precision heartbeat interval estimation within short time windows [12]. Furthermore, considering the impact of individual body type and physiological variations on detection results, system optimization integrating machine learning for personalized classification and specialized scenarios (such as driver health monitoring) has also been extensively validated [13,14,15].

### 2.2. Machine Learning and Deep Learning

To overcome the limitations of traditional signal processing methods in dynamic and complex environments, an increasing number of studies have introduced machine learning and deep learning approaches to achieve more efficient non-contact HRV detection. In preliminary attempts, low-latency, real-time heartbeat detection was achieved by processing raw signals from 24 GHz continuous-wave radar using artificial neural networks [16]. Subsequently, vital sign monitoring systems based on millimeter-wave radar not only achieved separation of heartbeat and respiratory signals but also incorporated cardiac arrhythmia detection functionality, thus demonstrating the potential for disease prediction using multi-layer neural networks [17]. More recently, further research has achieved accurate estimation of HRV parameters using only 4 s of data by constructing deep networks with spectral representation modules and residual nested structures, thereby effectively reducing detection delay while ensuring high measurement precision [18].

However, from a technical perspective, existing methods still face several challenges: traditional filtering and matching algorithms tend to introduce significant errors when processing composite signals containing respiration, heartbeat, and other background interference. Additionally, while deep learning methods show potential for improving HRV extraction precision, their generalization capability has not been fully validated due to limitations in the scale and diversity of training datasets; furthermore, some systems fail to fully utilize phase information in radar signals during the target detection stage, leading to error accumulation problems in the signal separation process.

Given the limitations of current technical approaches, this research aims to propose an improved method that addresses key issues in signal processing. The proposed method achieves three-dimensional positioning of human targets using beamforming and MTI (Moving Target Indication) moving target detection, and effectively reduces errors in the HRV parameter extraction process through a Second-Order Spectral Sparse Separation Algorithm Using Lagrangian Multipliers. The next section will introduce in detail the theoretical foundation and implementation process of this method and validate its effectiveness in complex environments through comprehensive experiments.

## 3. Overview of HRV Measurement Principles and Algorithms

This section introduces the non-contact HRV (Heart Rate Variability) detection algorithm based on FMCW millimeter-wave radar, and provides a detailed explanation of the three-dimensional human body positioning method and a Second-Order Spectral Sparse Separation Algorithm Using Lagrangian Multipliers.

### 3.1. HRV Detection Principles and Algorithm Process

**Figure 1:**
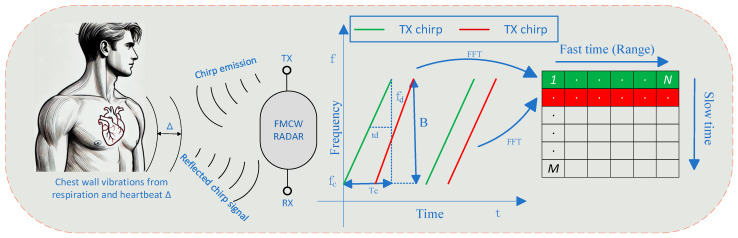
Principle of vital sign signal measurement using FMCW millimeter-wave radar.

FMCW millimeter-wave radar enables distance measurement between target objects and the radar through the emission of continuously frequency-modulated (chirp) electromagnetic wave signals. As illustrated in Figure 1, human respiration and heartbeat induce periodic vibrations of the thoracic wall ranging from micrometer to millimeter amplitude, resulting in subtle phase changes in the reflected signal. Via phase demodulation techniques, the corresponding vibration frequency and amplitude can be extracted, facilitating non-contact monitoring of heart rate variability (HRV).

Table 1 summarizes the typical parameters of thoracic wall vibrations induced by respiration and heartbeat, which are derived from related studies [19,20,21], but may differ slightly from the values reported in the literature. Due to the short wavelength of millimeter-wave signals, their phase changes exhibit exceptional sensitivity to minute displacements, thereby enabling effective detection of the subtle vibrations of the thoracic wall during respiration and heartbeat processes. FMCW radar concurrently provides distance measurement and micro-motion detection, while demonstrating robust anti-interference capabilities and real-time performance, thereby rendering FMCW millimeter-wave radar an ideal modality for non-contact HRV monitoring.

**Figure 2:**
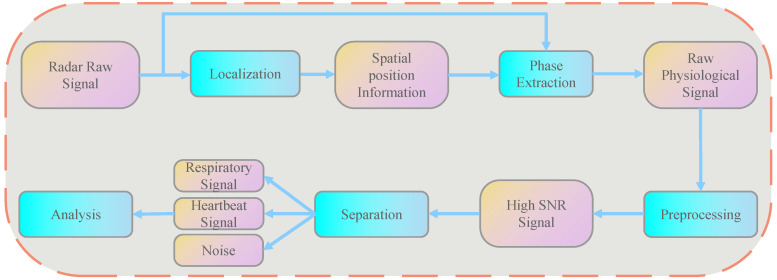
Signal processing algorithm flowchart.

Notably, the human body, as a target with finite dimensions, generates multiple reflection points during radar detection, resulting in a complex superposition of echo signals. This multi-point reflection poses significant challenges for traditional signal processing methods in accurately separating and extracting heart rate variability information. To address this challenge, we propose a comprehensive signal processing algorithm, with the overall flow chart presented in Figure 2, which systematically delineates the entire process from radar raw data to final HRV parameter analysis.

Specifically, following the acquisition of the radar’s original signal, a positioning algorithm initially localizes the target in three-dimensional space, yielding precise spatial information that facilitates phase extraction from the radar’s raw signal to obtain the original vital sign signal. Subsequently, our proposed Second Order Spectral Sparse Separation Algorithm Using Lagrangian Multipliers isolates the heartbeat signal from the processed high signal-to-noise ratio vital sign signal, enabling accurate HRV analysis. In the following sections, we present detailed descriptions of the target positioning algorithm and the Second-Order Spectral Sparse Separation Algorithm Using Lagrangian Multipliers.

### 3.2. Target Localization Algorithm

**Figure 3:**
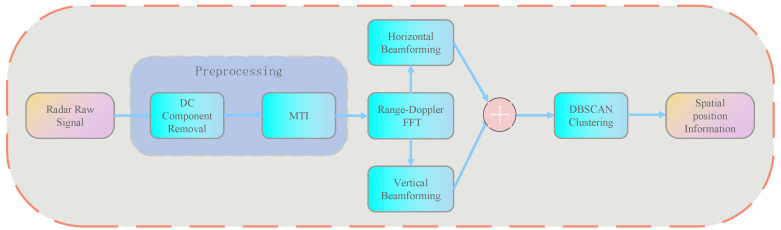
Target positioning algorithm flowchart.

Herein, we present a detailed description of the target localization algorithm implemented in this research, with its algorithmic flow illustrated in Figure 3. This method, based on raw data acquired from FMCW millimeter-wave radar, achieves accurate human target localization through a sequential pipeline of preprocessing, Range-Doppler transform, digital beamforming, three-dimensional energy fusion, and DBSCAN-based target clustering, thereby providing reliable target region information for subsequent HRV signal extraction.

#### 3.2.1. Signal Preprocessing and Spatial Spectral Analysis

To suppress static clutter, we first applied Moving Target Indication (MTI) filtering to each antenna signal. In the fast-time dimension, a Blackman-Harris window function was applied to the MTI-processed data, followed by Fast Fourier Transform (FFT), enabling spectral analysis in the range dimension. Subsequently, window function processing and FFT were similarly applied in the slow-time (chirp dimension) to obtain the Doppler spectrum, resulting in a two-dimensional Range–Doppler map.

To determine the target’s position in spatial angles, we utilized digital beamforming technology to reconstruct the processed data in the spatial domain. In this algorithm, different antenna combinations were strategically selected to achieve independent localization in both horizontal and vertical directions. After beamforming, energy normalization processing was performed to stabilize the relative energy distribution across different beams, yielding two sets of spatial energy distributions $E_{hor}(r,\theta_{x})$ and $E_{ver}(r,\theta_{z})$.

#### 3.2.2. Three-Dimensional Target Detection and Position Estimation

To achieve three-dimensional localization, these two spatial energy distributions were fused to construct a three-dimensional energy matrix whose elements are defined as follows:

(1) $$E_{3D}\left(i,j,k\right)=E_{hor}\left(r_{k},\theta_{x_{i}}\right)\times E_{ver}\left(r_{k},\theta_{x_{j}}\right),$$

where $r_{k}$ represents the k-th range dimension, and $\theta_{x_{i}}$ and $\theta_{x_{j}}$ correspond to the angular indices of the i-th horizontal beam and the j-th vertical beam. This matrix characterizes the distribution of reflection energy across different distance and angle regions, with targets typically manifesting as local areas of higher energy.

Since reflection energy is significantly affected by target distance, and to suppress noise and invalid points in the environment, $E_{3D}$ was normalized, and high-energy points satisfying the following condition were selected as candidate target points:

(2) $$E_{3D}(i,j,k)\geq \eta \cdot max\left\{E_{3D}(i,j,k)\right\},$$

where η denotes the energy threshold.

Subsequently, a density-based clustering algorithm, DBSCAN, was employed to perform cluster analysis on the candidate point set, effectively separating targets distributed in three-dimensional space, with each cluster corresponding to a candidate human target. For each clustering result, the frequency of occurrence of each point was counted, and the point with the highest frequency was selected as the representative position of the target.

Finally, the beam indices were converted to actual physical coordinates in the Cartesian system. The actual distance corresponding to each sampling point on the distance axis was calculated as follows:

(3) $$\left\{\begin{array}{c} x_{dis}=\frac{c\cdot N\cdot tan(\theta_{max})}{4B{\cdot N}_{beam}},\\ y_{dis}=\frac{c}{4B},\\ z_{dis}=\frac{c\cdot N\cdot tan(\theta_{max})}{4B{\cdot N}_{beam}}. \end{array}\right.$$

where c represents the speed of light, B denotes the bandwidth, N is the number of sampling points per chirp, $N_{beam}$ represents the number of beams, and $\theta_{max}$ indicates the maximum angle in the horizontal and vertical directions.

Therefore, for any index $\left(i,j,k\right)$ in the beam domain (where i and j represent the horizontal and vertical beam indices, respectively, and k denotes the distance index), its actual Cartesian coordinates were determined as follows:

(4) $$\left\{\begin{array}{c} x_{actual}=x_{dis}\left(i-\frac{N_{beam}}{2}\right),\\ y_{actual}=y_{dis}\cdot k,\\ z_{actual}=z_{dis}\left(j-\frac{N_{beam}}{2}\right). \end{array}\right.$$

**Figure 4:**
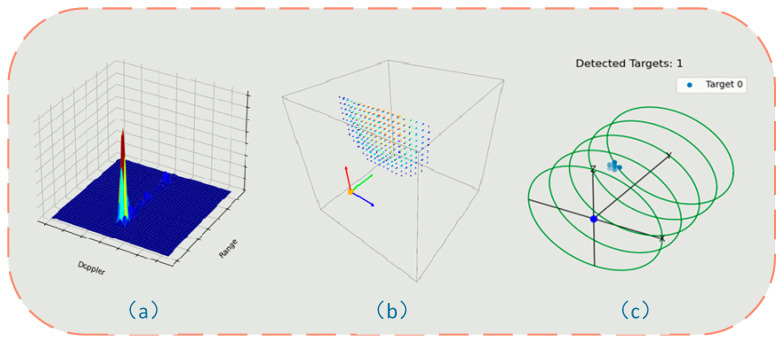
(**a**) Range–Doppler spectrum showing the distance-velocity characteristics of the target; (**b**) three-dimensional point cloud representation of high-energy scattering points extracted after matrix fusion; (**c**) target localization algorithm result showing the target’s actual location relative to the radar system.

Figure 4 illustrates the results of each process in the target localization algorithm.

### 3.3. Second-Order Spectral Sparse Separation Algorithm Using Lagrangian Multipliers

After obtaining the target spatial position information through robust target localization algorithms, the original FMCW radar signal is systematically transformed into a phase signal containing heartbeat information. The methodology comprises the following steps: Initially, Fast Fourier Transform (FFT) and digital beamforming are performed on each frame of the original signal to obtain range-angle domain data. Subsequently, phase information is extracted through the arctangent function based on the target spatial position information of the target cluster. To address phase jumps caused by 2π periodicity, phase unwrapping techniques are implemented through the addition of integer compensation terms to restore phase continuity. In the final stage, by calculating the variance of the phase signal and filtering out low-variance signals, static noise components are effectively removed, yielding a continuous phase sequence that encompasses not only the target’s respiratory movement and heartbeat information but also incorporates other minute motion artifacts. Based on this methodology, the original data is converted into high-quality phase signals, enabling the Second-Order Spectral Sparse Separation Algorithm Using Lagrangian Multipliers to further isolate fine heartbeat signals. The subsequent sections elucidate the key implementation and technical details of the Second-Order Spectral Sparse Separation Algorithm Using Lagrangian Multipliers in heartbeat signal extraction.

#### 3.3.1. Establishing Respiratory and Heartbeat Models

Utilizing the aforementioned method, high-quality phase signals containing both respiratory and heartbeat information were acquired. As demonstrated in Table 1, the amplitude of respiratory signals (~1–12 mm) significantly exceeds that of heartbeat signals (~0.01–0.2 mm), thereby causing substantial interference with the latter. Consequently, separating these two physiological signals has been a critical issue in related research. Traditional filtering methods primarily rely on frequency domain characteristics, while differential methods process signals by amplifying amplitude variations. However, both approaches exhibit inherent limitations and fail to fully elucidate the deeper distinctions between these physiological signals. To address these limitations, the present study further explores the fundamental differences between respiratory and heartbeat signals through rigorous mathematical modeling.

(a) With respect to respiratory signals, Antonio Albanese et al. developed a simulation model by dividing respiration into inhalation and exhalation phases [22], described by the following mathematical formulation:

(5) $$\left.x_{b}(t)=\left\{\begin{array}{l} \frac{-A_{r}}{T_{i}\cdot T_{e}}\cdot t^{2}+\frac{A_{r}\cdot T_{r}}{T_{i}\cdot T_{e}}\cdot t\;\;\;\;\;\;t\in [0,\;\;T_{i}]\\ \frac{A_{r}}{1-e^{-\frac{T_{e}}{\tau }}}\cdot \left(e^{-(\frac{\left(t-T_{i}\right)}{\tau })}-e^{-(\frac{T_{e}}{\tau })}\right)\;\;\;\;t\in [T_{i},\;\;T_{r}] \end{array}\right.\right.$$

where $\left[0,\;T_{i}\right]$ represents the inhalation phase, $\left[T_{i},\;T_{r}\right]$ represents the exhalation phase, and $T_{i}$ and $T_{e}$ denote the duration of inhalation and exhalation phases, respectively. $T_{r}$ indicates the total duration of the respiratory cycle, and $A_{r}$ represents the amplitude of respiration. The respiratory frequency can be calculated as $fr=2\pi /T_{r}$. Τ is the time constant of the respiratory curve, which determines the rate of filling and emptying of pulmonary gases. This modeling approach was subsequently adopted in studies by Sha Yuan and Yuki Iwata [18,23].

**Figure 5:**
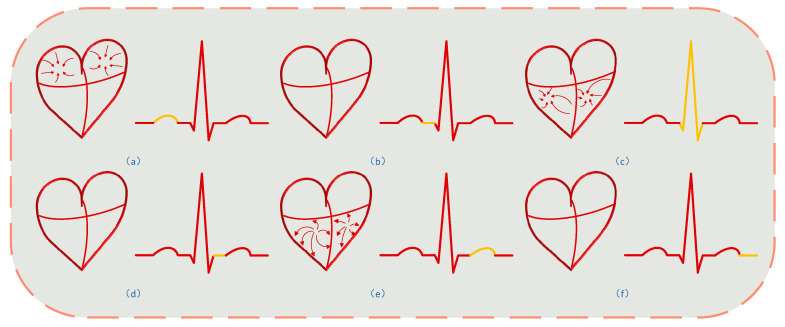
(**a**) Atrial contraction process, where blood flows from the atrium to the ventricle, corresponding to the P wave of the electrocardiogram; (**b**) the process after atrial contraction, where the ventricle prepares for contraction, corresponding to the PR interval of the electrocardiogram; (**c**) the ventricular contraction process, where blood flows from the ventricle to the lungs and body, corresponding to the QRS complex of the electrocardiogram; (**d**) the beginning of ventricular repolarization, where the ventricle prepares for the next contraction, corresponding to the ST interval of the electrocardiogram; (**e**) the ventricular repolarization process, where the ventricle returns to resting state, preparing for the next contraction, corresponding to the T wave of the electrocardiogram; (**f**) the period after ventricular repolarization, where the heart’s electrical activity enters a resting state, preparing for the next cardiac contraction, corresponding to the TP interval of the electrocardiogram.

(b) Building upon this conceptual framework, after stage-wise analysis of electrocardiogram signals and interpretation of the corresponding cardiac mechanical dynamics (as shown in Figure 5), we developed a five-stage model for heartbeat signals, governed by the following equation:

(6) $$x_{h}(t)=\left\{\begin{array}{ll} -\frac{K_{p}}{T_{p}^{2}}t^{2}+\frac{2K_{p}}{T_{p}}t & t\in [0,T_{p}]\\ K_{p} & t\in [T_{p},T_{PR}]\;\\ K_{QRS}e^{-\frac{(t-T_{QRS}{)}^{2}}{2\pi_{QRS}^{2}}} & t\in [T_{PR},T_{QRS}]\\ K_{QRS} & t\in [T_{QRS},T_{ST}]\\ K_{QRS}\frac{1-e^{-\lambda_{T}(t-T_{ST})}-e^{-\lambda_{T}(T_{RR}-T_{ST})}}{1-e^{-\lambda_{T}(T_{RR}-T_{ST})}} & t\in [T_{ST},T_{RR}] \end{array}\right.$$

where $K_{p}$ represents the atrial displacement amplitude constant; $K_{QRS}$ denotes the ventricular displacement amplitude constant; $\sigma_{QRS}=\sqrt{\frac{{\left(T_{PR}-T_{QRS}\right)}^{2}}{2\ln(\frac{K_{QRS}}{K_{P}})}}$ is the ventricular contraction rate; and $\lambda \geq \frac{5}{T_{RR}-T_{ST}}$ is the ventricular relaxation rate.

#### 3.3.2. Second-Order Spectral Sparse Separation Algorithm Using Lagrangian Multipliers for Separating Heartbeat Signals

In the preceding section, we utilized the target positioning algorithm results to facilitate the conversion of original FMCW radar data into phase signals, comprising respiratory signals, heartbeat signals, and human micro-motion noise, expressed as follows:

(7) $$y(t)=x_{b}(t)+x_{h}(t)+e(t)$$

where $x_{b}(t)$ represents the respiratory signal, $x_{h}(t)$ denotes the heartbeat signal, and e(t) corresponds to noise. It should be noted that both $x_{b}(t)$ and $x_{h}(t)$ are quasi-periodic signals with periods that may exhibit slight temporal variations. We assume that body movements introduce minimal oscillations, rendering $x_{b}(t)$ and $x_{h}(t)$ sparse in the spectral domain [11]. Let $\lambda_{bi}$ and $\lambda_{hj}$ denote the i-th eigenvalues of $x_{b}(t)$ and $x_{h}(t)$, respectively, arranged in descending order following eigenvalue decomposition. According to compressed sensing theory, the original signal can be reconstructed using the first n dominant eigenvalues. Furthermore, as observed in the stage-by-stage simulated waveforms presented in Section 3.3.1, the heartbeat signal exhibits more pronounced rising and falling structures compared to the respiratory signal, which can be approximated as a step function. By computing the second derivative of y(t), these fine step structures can be more effectively extracted. Consequently, we formulate the objective function to be minimized as follows:

(8) $$J(x_{b},x_{h},n)=\alpha_{b}\sum_{i=n+1}^{N}{\lambda_{bi}}^{2}+\alpha_{h}\sum_{j=n+1}^{N}{\lambda_{hj}}^{2}+\beta {\left\|\frac{d^{2}y(t)}{dt^{2}}-x_{h}(t)\right\|}_{2}^{2}+$$

where $\lambda_{bi}={eig}_{i}(R_{b})$, $R_{b}=\frac{1}{K}H\left(x_{b}\left(t\right)\right)H^{T}\left(x_{b}\left(t\right)\right)=U_{b}\Lambda_{b}U_{b}^{T};\lambda_{hj}={eig}_{j}\left(R_{h}\right),R_{h}=\frac{1}{K}H\left(x_{h}\left(t\right)\right)H^{T}\left(x_{h}\left(t\right)\right)=U_{h}\Lambda_{h}U_{h}^{T};\;{eig}_{i}\left(\cdot \right)$ represents the i-th eigenvalue of the covariance matrix $R\left(\cdot \right)$ arranged in descending order; $H\left(\cdot \right)$ denotes the operation of constructing a Hankel matrix; U=[u1,u2,…,uN] is the eigenvector matrix; K is the normalization coefficient, numerically equivalent to the number of columns in the Hankel matrix; N denotes the total number of decomposed eigenvalues; and $\alpha_{b}$ and $\alpha_{h}$ are hyperparameters that regulate the sparsity constraints. In this objective function, the first and second terms enforce sparsity of respiratory and heartbeat signals in the frequency domain, the third term preserves the integrity of fine step structures extracted via second-order differentiation, while the fourth term represents the data fidelity constraint.

Following the formulation of the objective function, our goal is to separate $x_{b}(t)$ and $x_{h}(t)$ such that the objective function is minimized. We fix n and employ the Lagrangian multiplier method to iteratively update $x_{b}(t)$ and $x_{h}(t)$. Initially, we establish the following constraints:

(9) $$\left\{\begin{array}{c} g_{bi}(x_{b}(t),\lambda_{bi})=\lambda_{bi}-\frac{1}{K}{\left\|H^{T}(x_{b}(t))\cdot u_{bi}\right\|}_{2}^{2}=0\\ g_{hj}(x_{h}(t),\lambda_{hj})=\lambda_{hj}-\frac{1}{K}{\left\|H^{T}(x_{h}(t))\cdot u_{hj}\right\|}_{2}^{2}=0 \end{array}\right.$$

Subsequently, we construct the Lagrangian function:

(10) $$L\left(x_{b},x_{h},\lambda_{b},\lambda_{h},u_{b},u_{h},\mu_{b},\mu_{h}\right)=J(x_{b},x_{h},n)+G\left(x_{b},x_{h},\lambda_{b},\lambda_{h},u_{b},u_{h}\right)$$

where $G\left(x_{b},x_{h},\lambda_{b},\lambda_{h},u_{b},u_{h}\right)=\sum_{i=1}^{N}\mu_{bi}\cdot g_{bi}(x_{b}(t),\lambda_{bi})+\sum_{j=1}^{N}\mu_{hj}\cdot g_{hj}(x_{h}(t),\lambda_{hj})$.

Having formulated the Lagrangian function, we proceed with the step-by-step parameter updates as follows:

(1). Fixing all other variables, we set the partial derivatives of the Lagrangian function with respect to $\lambda_{b}$ and $\lambda_{h}$ equal to zero, yielding the following update formulas:

(11) $$\left\{\begin{array}{c} \frac{\partial L}{\partial \lambda_{bi}}=2{\alpha_{b}\lambda }_{bi}+\mu_{bi}=0\\ \frac{\partial L}{\partial \lambda_{hj}}=2{\alpha_{h}\lambda }_{hj}+\mu_{hj}=0 \end{array}\right.\mathrm{and}\;\left\{\begin{array}{c} \lambda_{bi}^{\left(k+1\right)}=-\frac{\mu_{bi}^{\left(k\right)}}{2\alpha_{b}}\\ \lambda_{hj}^{\left(k+1\right)}=-\frac{\mu_{hj}^{\left(k\right)}}{2\alpha_{h}} \end{array}\right.$$

(2). Update $u_{b}$ and $u_{h}$ through covariance matrix decomposition:

(12) $$\left\{\begin{array}{c} R_{b}^{\left(k+1\right)}=\frac{1}{K}H(x_{b}^{\left(k\right)}(t))H^{T}(x_{b}^{\left(k\right)}(t))\\ R_{b}^{\left(k+1\right)}u_{bi}^{\left(k+1\right)}=\lambda_{bi}^{\left(k+1\right)}u_{bi}^{\left(k+1\right)} \end{array}\right.$$

This procedure yields $u_{bi}^{\left(k+1\right)}$, and analogously, $u_{hj}^{\left(k+1\right)}$ can be derived.

(3). Reconstruct $x_{b}(t)$ and $x_{h}(t)$ utilizing the updated $\lambda_{b}$, $\lambda_{h}$, $u_{b}$ and $u_{h}$:

First, compute the right singular vector:

(13) $$v_{bi}^{\left(k+1\right)}=\frac{1}{\sqrt{K\lambda_{bi}^{\left(k+1\right)}}}H^{T}(x_{b}^{\left(k\right)}(t))u_{bi}^{\left(k+1\right)}$$

Subsequently, reconstruct the Hankel matrix:

(14) $$H_{r}\left(x_{br}^{\left(k+1\right)}\left(t\right)\right)=\sum_{i=1}^{N}\sqrt{K\cdot \lambda_{bi}^{\left(k+1\right)}}\cdot u_{bi}^{\left(k+1\right)}\cdot v_{bi}^{\left(k+1\right)}$$

Finally, perform the inverse Hankel operation (diagonal averaging) to reconstruct $x_{b}(t)$:

(15) $$x_{br}^{\left(k+1\right)}(t)=\frac{1}{count(t)}\sum_{i+j=t+1}H_{r}\left(i,j\right)$$

where count(t) represents the number of elements $H_{r}\left(i,j\right)$ satisfying i+j=t+1, and $x_{hr}^{\left(k+1\right)}(t)$ is derived analogously.

(4). Set the partial derivatives of the Lagrangian function with respect to $x_{b}(t)$ and $x_{h}(t)$ equal to zero to determine the updated expressions:

(16) $$\frac{\partial L}{\partial x_{b}\left(t\right)}=-2\gamma \left(y\left(t\right)-x_{b}\left(t\right)-x_{h}\left(t\right)\right)-\frac{2}{K}\sum_{i=1}^{N}(\mu_{bi}\cdot D_{bi}^{T}\cdot S_{bi})=0$$

This yields

(17) $$x_{b}^{\left(k+1\right)}(t)=y(t)-x_{h}^{\left(k\right)}(t)-\frac{1}{K\gamma }\sum_{i=1}^{N}\left(\mu_{bi}^{\left(k\right)}\cdot D_{bi}^{T}\cdot S_{bi}^{\left(k+1\right)}\right)$$

where $S_{bi}^{\left(k+1\right)}=H^{T}(x_{b}^{\left(k\right)}(t))u_{bi}^{\left(k+1\right)},\;D_{bi}^{T}=\frac{\partial S_{bi}}{\partial x_{b}\left(t\right)}$. Analogously, the update formula for $x_{h}(t)$ can be derived:

(18) $$x_{h}^{\left(k+1\right)}(t)=\frac{\gamma \left(y\left(t\right)-x_{b}^{\left(k\right)}(t)\right)+\beta \frac{d^{2}y(t)}{dt^{2}}+\frac{1}{K}\sum_{j=1}^{N}\left(\mu_{hj}^{\left(k\right)}\cdot D_{hj}^{T}\cdot S_{hj}^{\left(k+1\right)}\right)}{\gamma +\beta }$$

where $S_{hj}^{\left(k+1\right)}=H^{T}(x_{h}^{\left(k\right)}(t))u_{hj}^{\left(k+1\right)},D_{hj}^{T}=\frac{\partial S_{hj}}{\partial x_{h}\left(t\right)}$.

(5). Update $\mu_{b}$ and $\mu_{h}$:

(19) $$\left\{\begin{array}{c} \mu_{bi}^{\left(k+1\right)}=\mu_{bi}^{\left(k\right)}+\rho (\lambda_{bi}^{\left(k+1\right)}-\frac{1}{K}{\left\|S_{bi}^{\left(k+1\right)}\right\|}_{2}^{2})\\ \mu_{hj}^{\left(k+1\right)}=\mu_{hj}^{\left(k\right)}+\rho (\lambda_{hj}^{\left(k+1\right)}-\frac{1}{K}{\left\|S_{hj}^{\left(k+1\right)}\right\|}_{2}^{2}) \end{array}\right.$$

Repeat the aforementioned five steps iteratively until the algorithm converges, with the convergence criterion defined as

(20) $$\left\{\begin{array}{c} {\delta_{b}=\left\|x_{br}^{\left(k+1\right)}-x_{b}^{\left(k+1\right)}(t)\right\|}_{2}^{2}<\varepsilon \\ {\delta_{h}=\left\|x_{hr}^{\left(k+1\right)}-x_{h}^{\left(k+1\right)}(t)\right\|}_{2}^{2}<\varepsilon \end{array}\right.$$

### 3.4. HRV Estimation

After extracting the heartbeat waveform, the precise temporal location of each cardiac cycle can be determined by identifying the peaks in the signal. The interbeat interval (IBI) is derived by calculating the temporal distance between consecutive cardiac cycles. This IBI sequence subsequently enables the evaluation of heart rate variability (HRV) parameters.

(21) $$MeanIBI=\frac{1}{N_{IBI}}\sum_{i=1}^{N_{IBI}}IBI(i)$$

where $N_{IBI}$ represents the total number of interbeat intervals in the measurement period.

The second metric, root mean square of successive differences (RMSSD), quantifies beat-to-beat variations in IBI and is calculated as

(22) $$RMSSD=\sqrt{\frac{1}{N_{IBI}-1}\sum_{i=2}^{N_{IBI}}{\left(IBI(i)-IBI(i-1)\right)}^{2}}$$

The third metric, standard deviation of normal-to-normal intervals (SDNN), characterizes the overall variability of IBIs and is defined as

(23) $$SDRR=\sqrt{\frac{1}{N_{IBI}}\sum_{i=2}^{N_{IBI}}{\left(IBI(i)-\bar{IBI}\right)}^{2}}$$

where $\bar{IBI}$ denotes the mean value of all interbeat intervals.

The fourth metric, pNN50, quantifies the percentage of successive interbeat intervals differing by more than 50 milliseconds, formulated as

(24) $$pNN50=\frac{\sum_{i=2}^{N_{IBI}}1(\left|IBI(i)-IBI(i-1)\right|>50)}{N_{IBI}}$$

where **1**(·) represents the indicator function.

## 4. Experimental Evaluation

This section presents a comprehensive evaluation of the proposed algorithm, including experimental methodology, performance analysis, and comparative assessment with state-of-the-art approaches.

### 4.1. Methodology

**Figure 6:**
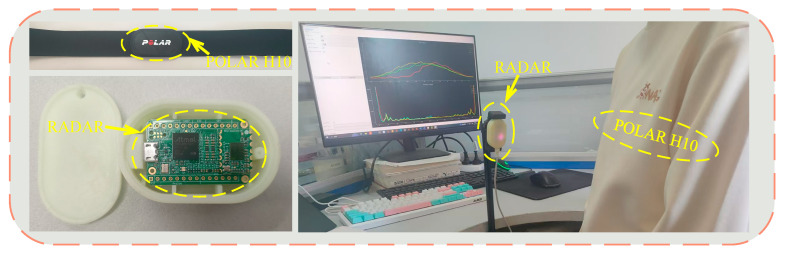
Experimental setup.

As illustrated in Figure 6, we conducted experiments using the Infineon BGT60TR13C millimeter-wave radar in an indoor environment with static-clutter interference. To obtain reference heartbeat signals, a Polar H10 heart-rate monitor—selected for its negligible electromagnetic impact on the radar—was used simultaneously with the radar. A total of 10 healthy participants (5 males and 5 females), aged 20–30 years, were recruited without restrictions on height or weight to enhance generalizability. Section 4.1 specifies the radar–subject geometry: four nominal distances (0.30, 0.50, 0.75, and 1.00 m) and three azimuth orientations relative to the radar boresight (0°, 15°, and 30°). A clear line of sight to the anterior chest surface was maintained throughout the experiments (no rigid obstructions; normal indoor clothing permitted, with metallic accessories over the chest removed). The Polar H10 ECG stream was sampled at 130 Hz; ECG and radar data were time-stamped on the same host and synchronized offline using a standard cross-correlation-based alignment between ECG R-peak and radar-derived beat events prior to evaluation. To further evaluate the performance of the proposed algorithm, we compared it with traditional bandpass filter methods using differential techniques [6] and advanced HRV estimation techniques [11]. The former eliminates respiratory interference by subtracting the envelope of the respiratory signal.

### 4.2. Overall Performance

**Figure 7:**
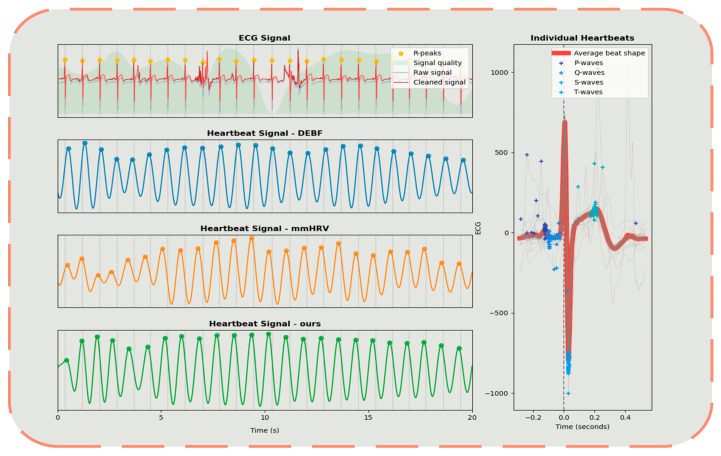
Signal waveform diagram.

Figure 7 illustrates the comparative results of heartbeat signals and ECG signals extracted using our proposed method, with the ECG signal obtained from a Polar H10 heart rate monitor serving as the reference for performance comparison. The left panel displays heartbeat signal waveforms extracted via three different methods: the DEBF method, the mmHRV technique, and our proposed Second-Order Spectral Sparse Separation Algorithm Using Lagrangian Multipliers. Visually, our method demonstrates significant advantages in terms of accuracy and smoothness, and exhibits superior correspondence with the peak positions of the ground truth (ECG signal). The right panel presents individual heartbeat analysis, distinctly revealing the regular characteristics of ECG signals. Compared to both traditional methods (DEBF) and state-of-the-art methods (mmHRV), our method not only identifies peak positions with greater precision but also provides enhanced signal quality stability. In subsequent quantitative analysis, our method consistently outperforms both DEBF and mmHRV methods across three key metrics: signal quality score, feature point positioning accuracy, and noise immunity. These results substantiate that the proposed algorithm exhibits high accuracy, technological advancement, and significant practical potential in the field of non-contact monitoring.

Table 2 shows the overall inter-beat interval (IBI) estimation accuracy of the proposed algorithm compared to traditional bandpass filter methods and advanced HRV estimation techniques. This experiment involved multiple participants undergoing multiple measurements under identical time and conditions, with results subsequently averaged.

The data presented in Table 2 demonstrate performance differences between the ECG method (as reference standard), DEBF, mmHRV, and our proposed method across multiple HRV metrics. Based on a comprehensive analysis of the experimental results, our method demonstrates significant advantages in heart rate variability estimation across all metrics. For the Mean IBI measurement, our method achieved a mean absolute error of 10.80 ms, representing error reductions of 50.25% compared to DEBF (21.71 ms) and 59.34% compared to mmHRV (26.56 ms). For the root mean square of successive differences (RMSSD) metric, our method’s mean absolute error was 25.80 ms, representing improvements of 41.12% compared to DEBF (43.82 ms) and 50.50% compared to mmHRV (52.12 ms). For the standard deviation of RR intervals (SDRR) metric, our method’s mean absolute error was 20.98 ms, showing reductions of 37.76% compared to DEBF (33.71 ms) and 38.31% compared to mmHRV (34.01 ms). For the percentage of successive NN intervals that differ by more than 50 ms (pNN50) metric, our method’s mean absolute error was 5.71%, decreasing errors by 47.57% compared to DEBF (10.89%) and 49.82% compared to mmHRV (11.38%). These results indicate that the proposed method achieves superior precision in heart rate variability estimation compared to existing approaches.

**Figure 8:**
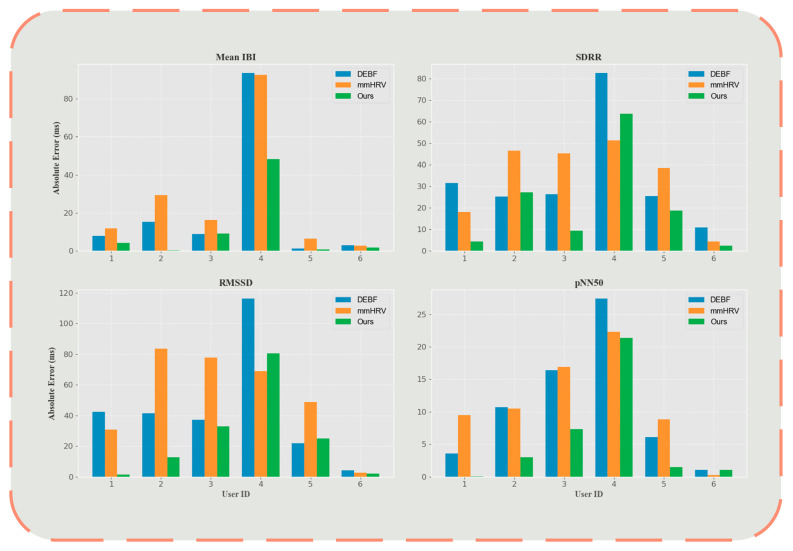
Absolute error bar chart.

Figure 8 intuitively illustrates the advantages of the proposed method. Across all four metrics, our approach achieves significantly lower absolute error than both DEBF and mmHRV. Specifically, for the Mean IBI metric, the error achieved by our method for Users 1, 2, 3, and 5 is substantially lower than the errors of DEBF and mmHRV. Notably, for Users 5 and 6, our method yields the smallest error, indicating enhanced consistency and stability under multi-user conditions. Regarding SDRR, although all methods exhibit larger errors for User 4, our approach attains lower errors overall—especially for Users 2 and 6—demonstrating a significant reduction compared to DEBF and mmHRV. Results for RMSSD and pNN50 further corroborate the superiority of our method: for Users 1, 2, and 3, the errors are smaller than those of traditional methods, underscoring its enhanced accuracy.

It is noteworthy that Subject 4 represented a significant outlier for all methods, with consistently higher errors across various metrics, potentially attributable to this subject’s unique physiological characteristics or specific data collection conditions. After excluding this anomalous case from the analysis, the performance gap between methods further widened, demonstrating that our algorithm exhibits higher accuracy and reliability under typical physiological conditions.

### 4.3. Impact of Distance and Angle

**Figure 9:**
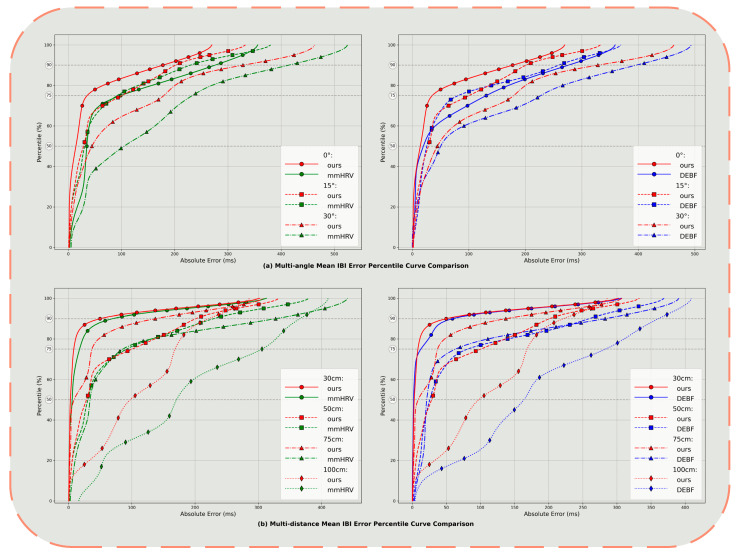
Empirical cumulative distribution function of Mean IBI estimation error at multiple angles and distances.

In this section, we analyze the impact of distance and angle between subjects and millimeter-wave radar on estimation error. Participants were positioned facing the device while seated at four different distances (30, 50, 75, 100 cm) and three different angles (0°, 15°, 30°). The empirical cumulative distribution function (CDF) of the Mean IBI estimation error is presented in Figure 9.

Figure 9a illustrates the cumulative distribution of Mean IBI estimation errors for each method at angles of 0°, 15°, and 30°. When subjects directly face the radar (0°), our method achieves an error of approximately 14 ms at the median point (50th percentile), while mmHRV and traditional bandpass filter methods exhibit errors of approximately 35 ms and 20 ms, respectively. As the angle deviates from front-facing, errors for all three methods increase, but with significant differences in error magnitude: At 15°, the median error of our method only rises to about 34 ms, which is similar to mmHRV and traditional bandpass filter methods; however, under the more extreme condition of 30°, our method’s median error is maintained at around 44 ms, while comparison methods approach 95 ms and 50 ms for mmHRV and bandpass filter, respectively. At the 90th percentile (90% cumulative probability position), our method demonstrates values of approximately 176 ms, 193 ms, and 326 ms at 0°, 15°, and 30°, respectively, significantly lower than mmHRV’s 276 ms, 230 ms, 415 ms and the bandpass filter’s 276 ms, 258 ms, 397 ms. These results indicate that in scenarios where signal amplitude is reduced due to orientation offset, our method not only excels in typical error levels (median) but also demonstrates superior suppression capability for tail errors (high percentile).

Figure 9b illustrates the performance degradation trend as the distance between subjects and the radar increases from 30 cm to 100 cm. At 30 cm, all three methods exhibit low errors—our method’s median error is approximately 3 ms, while mmHRV and bandpass filter methods show 6 ms and 3 ms, respectively. However, as the distance increases to 50 cm and 75 cm, the error curves shift rightward, with mmHRV’s median error approaching 35 ms at 75 cm, and the bandpass method exceeding 20 ms, while our method only increases to about 5 ms, indicating a more gradual performance decline. Even at the maximum tested distance of 100 cm, our method still maintains the median error within 92 ms, with the 95th percentile below 265 ms, while mmHRV’s corresponding value exceeds 395 ms, and the bandpass filter method similarly approaches 395 ms. These findings demonstrate that our method is more robust to signal-to-noise ratio (SNR) reduction related to distance and can maintain stable, accurate heartbeat interval estimation over a wider spatial range.

**Figure 10:**
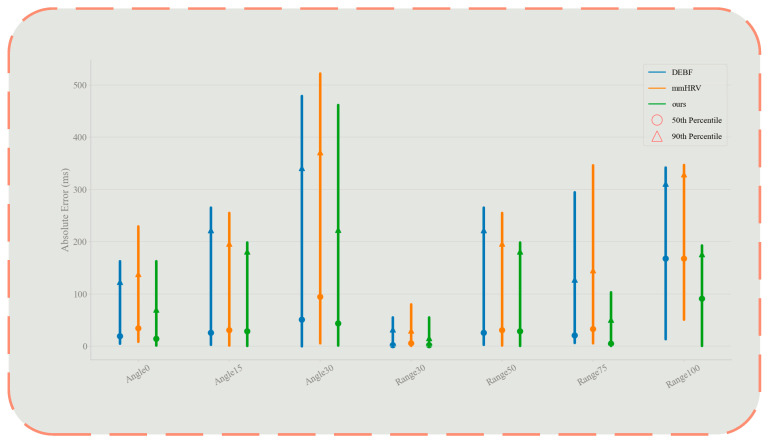
Comparative confidence interval charts of mean absolute IBI errors under multiple angles and distances.

To quantitatively demonstrate the performance differences among various methods under different conditions, Figure 10 presents comparative confidence interval charts of mean absolute IBI errors under multiple angles and distances, where dots represent the upper limit of 50% confidence intervals, triangles represent the upper limit of 90% confidence intervals, and line segments represent error bands. The results indicate that as the angle increases and distance extends, the errors of all methods demonstrate an upward trend; however, our proposed method consistently exhibits a statistically significant advantage: In the angle experiment, even under the extreme 30° condition, the median error of our method remains significantly lower than all comparative methods, with the 90% confidence interval upper limit constrained below 250 ms. In the distance experiment, our method demonstrates negligible error margins at short distances, and maintains relatively small error ranges at 100 cm (90% confidence interval upper limit of approximately 300 ms), whereas the error bands of comparative methods significantly expand (approaching or exceeding 400 ms), with differences particularly pronounced at the 90% confidence level, thus substantiating the superior robustness of our proposed method.

In summary, regarding the two typical interference factors—angle offset and distance extension—our proposed method significantly outperforms existing mmHRV algorithms and bandpass filter-based baseline schemes: not only does it achieve lower estimation errors in common confidence intervals (50th–75th percentile), but it also effectively suppresses tail errors in high confidence intervals (90th–95th percentile). These results suggest that our method offers enhanced reliability and applicability for practical applications.

## 5. Discussion and Future Work

In this study, we proposed and validated a three-dimensional human positioning and iterative optimization method for heart rate variability (HRV) extraction based on frequency-modulated continuous wave (FMCW) millimeter-wave radar. Experimental results demonstrated that compared to traditional bandpass filter arrays and advanced mmHRV methods, our approach significantly reduced estimation errors under multi-distance and multi-angle conditions, achieving high-precision separation and robust extraction of respiratory and heartbeat signals. Although the proposed method performed excellently in resting states, several limitations remain, including the relatively controlled experimental environments, manually tuned algorithm parameters, and susceptibility to interference from subtle human body movements. Future work will focus on the following. 

**Comparison with advanced methods and dataset availability.** Recent deep-learning approaches have shown promise for radar-based HRV estimation. However, a fair, reproducible head-to-head comparison is currently hindered by the absence of a publicly validated, sufficiently large radar–ECG/HRV dataset. Our pipeline is model-based and does not require supervised training, which enables stable operation with limited data, whereas deep networks typically demand diverse, large-scale cohorts to generalize reliably. As part of future work, we will curate and release a standardized radar–HRV dataset with unified acquisition protocols (resting, light speech/typing, mild movement) and will implement representative deep architectures under identical preprocessing and evaluation metrics as in Section 4, so that rigorous benchmarking against advanced deep learning methods becomes possible.

**Evaluation in dynamic and realistic scenarios.** While this study intentionally targeted low-motion clinical settings (e.g., bedridden, burn, ICU) to align with the intended application, we recognize that dynamic behaviors—such as speaking, light typing, mild postural changes, and slow walking—are common in real life and can degrade radar-based HRV estimation. In future work, we will expand the experimental protocol to include controlled mild-motion tasks and ambient perturbations (e.g., background human movement and airflow), and report subject-independent results under fixed radar placements as well as moderate angle/distance shifts. We will leverage the Moving Target Indication (MTI) stage already present in our pipeline to suppress gross body motion and, where needed, augment it with micro-motion artifact rejection. 

**Real-time latency and feasibility.** In this study, the pipeline was executed offline to prioritize estimation fidelity. Nevertheless, all stages—MTI, phase demodulation/unwrapping, respiratory suppression, peak detection, and HRV index computation—are compatible with a streaming implementation. In practice, the apparent delay is governed mainly by the analysis window required to produce stable HRV statistics: shorter windows yield faster updates but noisier estimates, whereas longer windows improve stability at the cost of responsiveness. Beat-to-beat indicators (e.g., IBI traces and instantaneous heart rate) can be refreshed continuously as peaks are detected, while aggregate HRV indices can be updated on a rolling basis. The Hankel-structured operations in our method introduce additional computation; as future work, we will explore incremental/low-rank updates and lightweight implementations to ensure practical real-time operation without compromising accuracy.

## Figures and Tables

**Table 1:** Respiration and heartbeat vibration frequencies and amplitudes.

Vital Signal	Frequency	Amplitude
Breathing Rate (Adults)	0.1–0.5 Hz	~1–12 mm
Heart Rate (Adults)	0.8–2.0 Hz	~0.01–0.2 mm

**Table 2:** HRV estimation using Mean IBI, RMSSD, SDRR, and pNN50 (partial).

Metrics	Methods		User ID					
			1	2	3	4	5	6
Mean IBI	Value (ms)	ECG	838.24	744.74	720.79	764.07	592.71	751.42
		DEBF	846.25	760.11	711.89	857.55	591.30	754.49
		mmHRV	850.09	774.11	737.20	856.58	599.05	754.29
		Ours	842.56	744.31	711.56	812.39	592.01	753.20
	Error (ms)	DEBF	8.01	15.37	**8.90**	93.49	1.41	3.07
		mmHRV	11.85	29.37	16.41	92.51	6.34	2.87
		Ours	**4.33**	**0.43**	9.23	**48.32**	**0.70**	**1.78**
SDRR	Value (ms)	ECG	31.06	37.58	24.65	21.14	7.67	27.81
		DEBF	62.64	62.81	51.02	103.76	33.22	38.73
		mmHRV	49.22	84.10	69.81	72.56	46.19	32.11
		Ours	26.60	10.36	34.01	84.82	26.43	25.41
	Error (ms)	DEBF	31.58	**25.22**	26.37	82.62	25.54	10.92
		mmHRV	18.16	46.51	45.16	**51.42**	38.52	4.30
		Ours	**4.46**	27.22	**9.36**	63.68	**18.76**	**2.40**
RMSSD	Value (ms)	ECG	30.79	22.19	12.46	21.25	7.26	25.89
		DEBF	73.09	63.52	49.69	137.34	29.11	29.99
		mmHRV	61.53	105.76	90.29	90.24	56.15	28.54
		Ours	32.26	9.45	45.35	101.85	32.31	23.86
	Error (ms)	DEBF	42.30	41.33	37.23	116.09	**21.86**	4.10
		mmHRV	30.74	83.57	77.83	**69.00**	48.90	2.65
		Ours	**1.47**	**12.74**	**32.89**	80.60	25.05	**2.03**
pNN50	Value (ms)	ECG	8.78	3.00	0.00	1.91	0.00	4.74
		DEBF	12.38	13.72	16.42	29.29	6.12	5.83
		mmHRV	18.24	13.50	16.91	24.22	8.83	4.49
		Ours	8.70	0.00	7.31	23.24	1.52	5.77
	Error (ms)	DEBF	3.61	10.72	16.42	27.39	6.12	1.09
		mmHRV	9.47	10.50	16.91	22.32	8.83	**0.25**
		Ours	**0.08**	**3.00**	**7.31**	**21.34**	**1.52**	1.03

## Data Availability

Data will be made available upon request.
